# The Efficacy of Magnetic Resonance Imaging and X-Ray in the Evaluation of Response to Radiosynovectomy in Patients with Hemophilic Arthropathy

**DOI:** 10.4274/MIRT.25

**Published:** 2011-08-01

**Authors:** Tamer Özülker, Filiz Özülker, Esin Derin, Mehmet Altun, Gönül Aydoğan, Emine Türkkan, Müjdat Adaş, Murat Tonbul, Tevfik Özpaçacı, Funda Sezgin, Hülya Değirmenci

**Affiliations:** 1 Okmeydanı Training Hospital, Nuclear Medicine, İstanbul, Turkey; 2 Fatih Sultan Mehmet Hospital, Department of Radiology, İstanbul, Turkey; 3 Okmeydanı Training Hospital, Department of Orthopedics, İstanbul, Turkey; 4 Bakırkoy Gynecology and Obstetrics Hospital, Department of Pediatric Hematology, İstanbul, Turkey; 5 Okmeydanı Training Hospital, Department of Child Health, İstanbul, Turkey; 6 Mimar Sinan University, Department of Biostatistics, İstanbul, Turkey; 7 Okmeydanı Training Hospital, Department of Radiology, İstanbul, Turkey

**Keywords:** Radiosynovectomy, hemophilic arthropathy, magnetic resonance imaging, therapy response

## Abstract

**Objective:** We aimed to assess the role of Magnetic Resonance Imaging (MRI) and X-Ray in the evaluation of response to radiosynovectomy (RS) in patients with hemophilic arthropathy.

**Material and Methods:** Eleven patients who suffered from hemophilic arthropathy with a mean age of 11.7 (range between 7-15) were included in this study. 148-185 MBq Yttrium 90 silicate (Y-90) was administered intraarticularly to ten knee joints and one patient was treated with intraarticular 74 MBq Rhenium 186 (Re-186) injection into his ankle. Before radiosynovectomy, plain anteroposterior and lateral X-rays of the target joints were obtained by standard technique. The follow-up MRI and X-ray studies of the patients were done 6 months after RS. Pettersson hemophilic arthropathy scales were utilized to stage the condition of the joints on plain X-ray and classification of the investigated joints on MRI were done according to Denver score. The clinical assessment of the efficacy of the RS was made with the comparison of the average bleedings before and after the intervention.

**Results:** During the 6-month follow-up period after RS, an improvement in number of hemarthrosis 75% or greater compared with the prior six months occurred in six joints (54.5%). The Pettersson scores worsened in 1/11 (9%), remained unchanged in 9/11 (81.8%), and improved in 1/11 (9%) joints. At the 6-month follow-up, the MRI score worsened in one (9%) and was unchanged in 10/11 joints (90.9%).

**Conclusion:** MRI is a more sensitive tool than plain radiography for evaluating and follow-up of joint disease in persons with hemophilia, but both methods don’t show correlation with the therapeutic response

**Conflict of interest:**None declared.

## INTRODUCTION

The most frequently encountered hemorrhages in hemophilia are intra-articular bleedings and hemophilic arthropathy that occurs consequently, remains one of the major complications of severe hemophilia. After intraarticular bleeding, absorption of blood breakdown products by the synovium causes inflammation and consequently a cycle of hemarthrosis–synovitis– hemarthrosis occurs. The major principle in treating hemophilic patients is to interrupt this cycle which cannot be broken by standard conservative measures, such as factor replacement and physiotherapy. To achieve this goal, this hypervascular and hypertrophic synovium should be destroyed by either operative synovectomy or radiosynovectomy (RS). RS has been used effectively for this purpose since 1952 and satisfactory results were obtained (1,2,3,4,5,6,7,8,9,10,11,33,34,35). The assessment of the therapy response of RS in patients with hemophilic arthropathy has been done by measuring the changes in mean number of hemorrhages, pain in the joints, range of motion (ROM) or by imaging studies like blood-pool imaging, plain X-ray and MRI. 

In this study we tried to compare the efficacies of plain radiography, MRI and clinical findings in the assessment of joint outcome in patients treated with RS for hemophilic arthropathy. 

## MATERIALS AND METHODS

Eleven patients (6 hemophilia A, 5 hemophilia B) who suffered from hemophilic arthropathy with a mean age of 11.7 (range between 7-15) were included in this study. Between January 2004 and December 2009 ten knee joints of ten patients were administered 148-185 MBq Yttrium 90 silicate (Y-90) (CIS, Gif-Sur-Yvette Cedex, France) intraarticularly and one patient was treated with intraarticular 74 MBq Rhenium 186 (Re-186) (CIS, Gif-Sur-Yvette Cedex, France) injection into his ankle. Patients who fulfilled the following prerequisites were included for radiosynovectomy application: (1) more than four hemorrhagic episodes in six months (2) persistent synovitis. All decisions were given in our hemophilic arthropathy council composed of an expert orthopedist, pediatric hematologists, a nuclear medicine specialist and a physiotherapist. All patients were admitted to the hospital and treated with factor replacement so as to raise the factor level of the patient to 80% the following morning and 50% for three days thereafter. Factor VIII (Hemofil M, Eczacibasi-Baxter, Istanbul, Turkey) or Factor IX (Immunine, Eczacibasi-Baxter) complexes were used for factor replacement. The effusion in the joint was evacuated before the injection of the radiocolloid. Intraarticular injection in ankle joint was done under fluoroscopic guidance. An empirically estimated dose of 148-185 MBq of Y-90 and 74 MBq of Re-186 was injected to the joint. After the injection, the needle was flushed with a small volume of saline and the needle was withdrawn. The joint was moved rapidly a few times to distribute the radiocolloid, after which a plaster of paris cast was applied for 72 hours. One hour after the RS, an image of the treated joint and the regional lymph nodes was made with a gamma camera to confirm the appropriate distribution of the radionuclide in the joint. Before RS, plain anteroposterior and lateral X-rays of the target joints were obtained by standard technique. MRI of the joints was performed with a magnetic resonance (Philips, Netherland) with field strength of 1.5 Tesla. The follow-up MRI and X-ray studies of the patients were done 6 months after RS. Evaluation of the imaging studies before and after RS were done by an experienced musculoskeletal radiologist (ED). Pettersson hemophilic arthropathy scales (Table 1) was utilized to stage the condition of the joints on plain X-ray and classification of the investigated joints on MRI were done according to Denver score (Table 2). The clinical assessment of the efficacy of the RS was made with the comparison of the average bleedings before and after the intervention.

Differences in scores of MRI, X-ray and bleeding frequency between the measurements done before and after the RS were examined for statistical significance using the Wilcoxon signed rank test. The results were considered significant when p value was <0.05.

The study is approved by the ethical review board of our hospital and informed consent is obtained from each participant.

## RESULTS

Eleven boys who had undergone RS for hemophilic arthropathy in eleven joints were included in the study. 

The Denver MRI and Pettersson X-ray scores were calculated for each joint prior to RS and at the evaluation done 6 months after RS.

During the 6-month follow-up period after RS, an improvement in number of hemarthrosis 75% or greater compared with the prior six months occurred in six joints (54.5%). In four joints (36.3%), there was not any change in bleeding frequency and in one joint (9%) the number of bleedings per month increased after the procedure. Four (36.3%) treated joints had a complete cessation of bleeding episodes after therapy during the 6-month follow-up.

The median number of bleedings into the target joints was 2.9±1.9 in the six months prior to the procedure and 1.1±1.3 in the following 6 months. This change in the number of bleedings was statistically significiant (p<0.05). The median WFH orthopedic joint score changed from 4.5±2.5 prior to the procedure to 4.8±3.4 at six months after RS. The median WFH pain score was 0.7±0.6 prior to the RS and 0.5±0.6 six months later.

**Figure 1:**
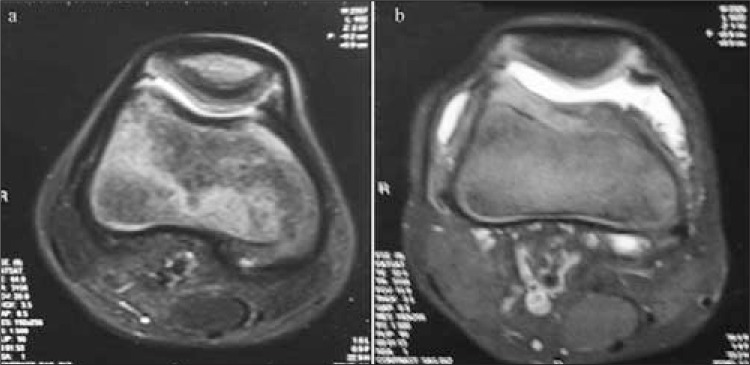
The axial fat-saturated T2 weighted MRI images before radiosynovectomyshow only minimal suprapatellar effusion (a), synovial hypertrophy,significant suprapatellar effusion and more than 50% cartilage loss 6months after radiosynovectomy (b)

**Figure 2:**
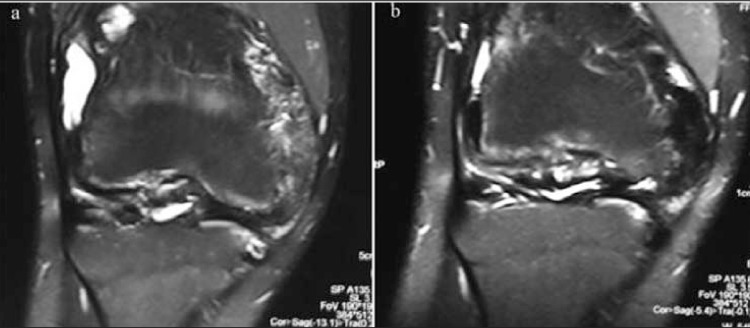
The coronal fat-saturated T2 weighted MRI images beforeradiosynovectomy show narrowing in joint space, synovial hypertrophy,moderate amount of effusion (a) and a decrease in the amount of effusion,subchondral hypointense areas and cartilage defects in the lateral sideand development of hypointensity due to hemosiderin pigment accumulationin the hypertrophic synovium at the medial side is observed in theimages obtained 6 months after radiosynovectomy (b)

**Figure 3:**
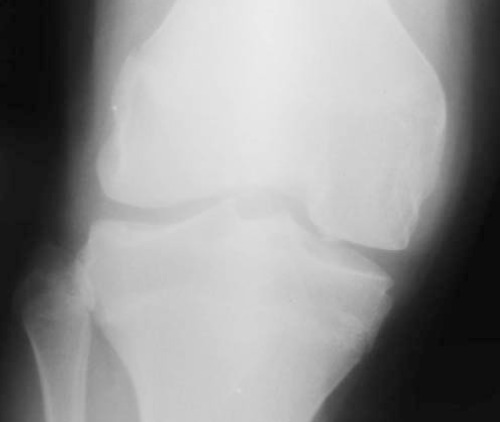
X-ray of the knee of the patient in Figure 2 shows narrowing inthe tibiofemoral joint space medially, increased subchondral sclerosis in thejoint spaces and minimal cortical irregularities in the medial condyle of the femur

The Pettersson scores worsened in 1/11 (9%), remained unchanged in 9/11 (81.8%), and improved in 1/11 (9%) joints. At the 6-month follow-up, the MRI score worsened in one (9%) (Figure 1) and did not show remarkable changes in 10/11 joints (90.9%) (Figure 2, 3). The median Pettersson score was 1.9±1.4 before RS and 1.9±1.3 at the 6-month follow-up. The median MRI scores were 8.8±3 and 9.6±0.9 respectively, initially and at the 6-month follow-up. The changes in the scores of MRI and X-ray between the measurements made before and after RS was not significant statistically (p>0.05). 

**Figure 4:**
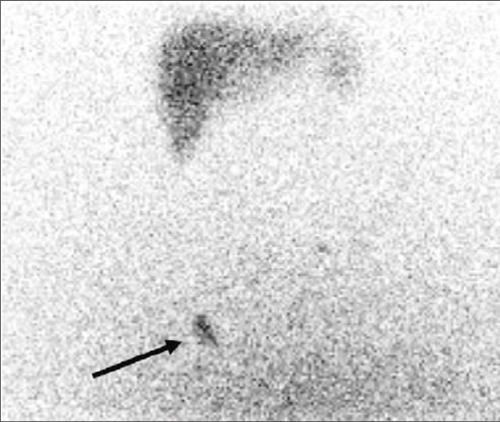
Unusual uptake of radicolloid in liver and right inguinal lymphnode due to extraarticular leakage of Re-186

We did not encounter any complications in patients but there were local lymph node and liver visualization in control scintigraphy of one patient, due to extra-articular leakage of Re-186 (Figure 4).

## DISCUSSION

In the present study, the frequency of hemarthroses decreased in 54.5% of patients after RS. This success rate is not as high as the previous studies which reported improvement from 75% to 90% after radiosynovectomy(2,3,4,5,6,7,8,9,10,11,33,34). This relatively low success rate might be explained with the high ratio of advanced disease in our patient group, as 10 out of 11 joints showed cartilage loss on pre-procedure MRI. There are reports indicating that the likelihood of a favourable response was higher in joints with no or little radiological damage, similar to the findings in patients with rheumatoid arthritis (10,11,12). It has been proposed that initial findings on MRI, in particular, measures of the severity of synovial hyperplasia, may be inversely related to clinical response to radiosynoviorthesis (13). In four joints (36.3%) no change was observed in the mean number of hemarthrosis, while in one joint deterioriation of clinical findings was seen. Although the success rate regarding the bleeding frequency seemed to be less than the literature findings, the median number of bleedings into the target joints dropped from 2.9±1.9 per month to 1.1±1.3 per month after the procedure and this decrease was statistically significant.

MRI and plain radiography are the major methods that have been utilized to document and follow the progression of joint damage prior to and following RS. Türkmen et al reported that blood-pool imaging with Tc-99m MDP might also be an objective means for monitoring therapy response in these patients (1). The most commonly used methods for radiological evaluation of hemophilic arthropathy are the Arnold–Hilgartner scale (13) and the Pettersson score (14), which are based on conventional radiography. There have been numerous MRI scoring methods proposed for evaluation of hemophilic arthropathy (13,16,17,18,19,20). Nuss et al designed the Denver scale, which was the first of these (13,20). In Denver scale and the Arnold–Hilgartner scale, the pathology is scored according to characteristic stages of development, thus following a progressive strategy, the score is determined by the most severe pathology in the joint. The additive methods like Pettersson radiographic score depends on summation of specific imaging findings so all findings affect the evaluation resulting in a higher sensitivity for detection of the disease progression (21). Although it is easy to use progressive scoring methods, it has been reported that additive methods make the discrimination between early and advanced arthropathy better (17,22).

In a study by Nuss et al, it has been reported that while MRI was superior to clinical examination and plain X-ray in identifying synovial hyperplasia and effusions, plain X-rays were found to be adequate to detect cysts, erosions and cartilage loss in joints with hemophilic arthropathy (23). In our patient group late stage findings that were not detected with plain X-ray, were effectively visualized on MRI. While 10 out of 11 joints showed cartilage loss detected by MRI , the scores on Pettersson scale were between 0-4 indicating early signs of degeneration (Table 3), so X-ray underestimated the joint damage as it did in the study made by Nuss et al (13). Funk et al reported that MRI score describes initial joints alterations more precisely and earlier than the Pettersson score (24). 

Our patient group was consisted of children with advanced stage arthropathy. The synovial activity in end-stage haemophilic joints goes on increasing despite dramatic bone and cartilage loss (23). It has been well known that, despite its clinical efficacy, RS does not prevent progressive severe joint degeneration in hemophilic joints as seen on both plain radiography and MRI (25,26,27,28,29,30,31,32) and our findings are also in congruence with this result as there were no significant difference between the mean scores of MRI and plain X-ray measurements before and after the procedure. 

In a study made among 78 hemophilic arthropathy patients, Corte-Rodriguez et al. categorized the variables with regard to the degree of improvement achieved after RS and they found that the number of episodes of hemarthrosis and the severity of pain were the variables associated with the greatest improvement (36). In the same study the tenth parameter, the WFH radiologic score, showed no improvement while the remaining nine parameters studied improved independently for each one of the intra-articular injections of the radioisotope. Our experience in this small patient group is also showed that clinical findings like bleeding frequency is better than imaging studies in the follow-up of the patients who underwent RS. 

The relatively low number of joints enrolled is a limitation the study. We think that further studies with larger series will be helpful to clarify the role of conventional imaging methods in the follow-up of joints treated with RS for hemophilic arthropathy. 

## CONCLUSION

RS is effective and safe in the treatment of chronical synovitis of children with hemophilia even at the later stages of the hemophilic arthropathy. MRI is a more sensitive tool than plain radiography for evaluating and follow-up of joint disease in persons with hemophilia, but both methods show weak correlation with the therapeutic response since despite the favourable clinical results obtained after RS, a progressive deterioration and irreversibility of the radiographic scores for the joints in most patients is observed. Observing the change in the number of bleeding episodes seems to be a more realistic way of following response to RS in patients with hemophilic arthropathy. 

## Figures and Tables

**Table 1:**
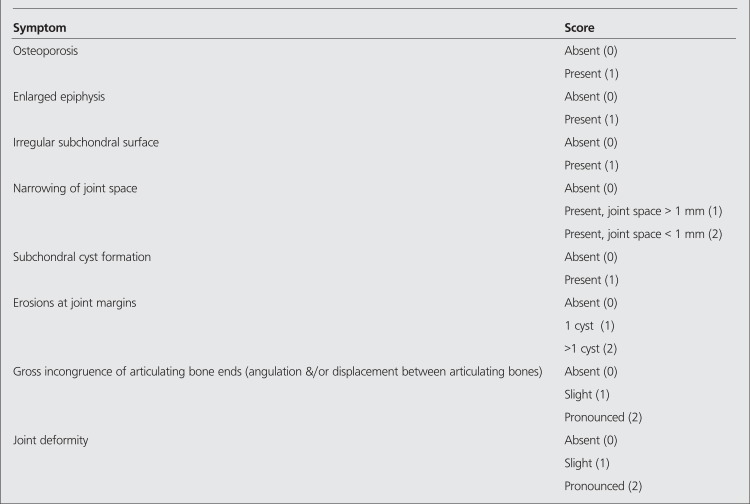
Pettersson scale

**Table 2:**
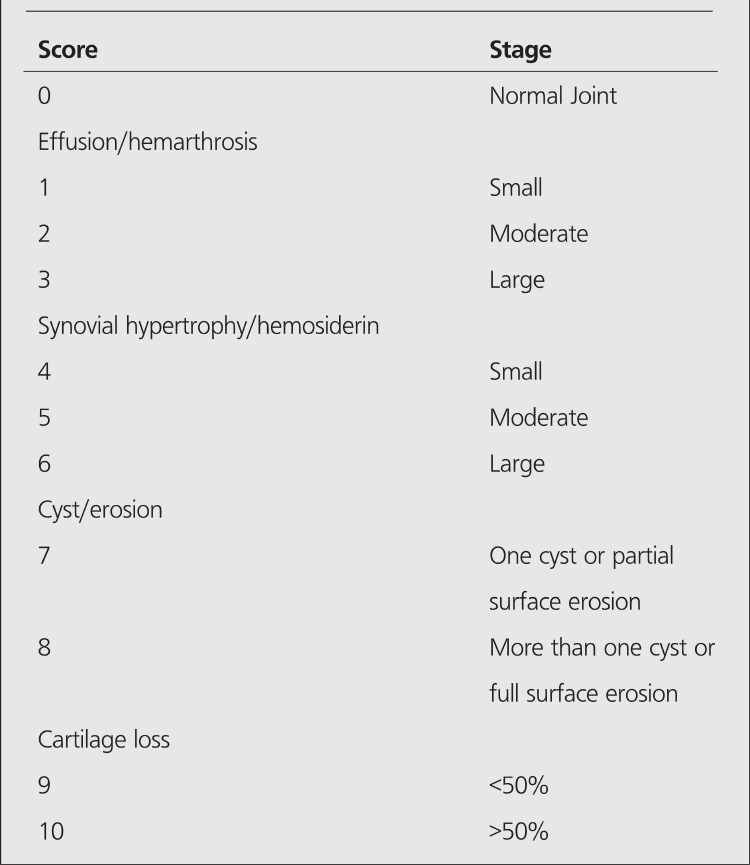
The Denver MRI scale classifying the arthropathy indifferent stages in relation to the most severe finding.The maximum score is 10

**Table 3:**
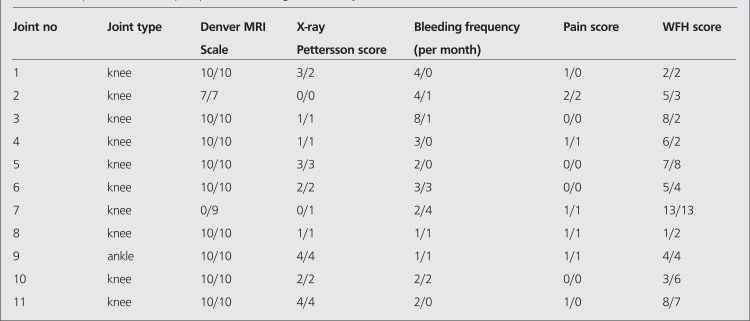
The pre- and 6-month post-procedure findings for the 11 joints studied
